# Sex bias in utero alters ovarian reserve but not uterine capacity in female offspring^†^

**DOI:** 10.1093/biolre/ioac208

**Published:** 2022-11-17

**Authors:** Annika V Geijer-Simpson, Haidee Tinning, Tiago H C De Bem, Ioannis Tsagakis, Alysha S Taylor, Laura Hume, Lisa M Collins, Niamh Forde

**Affiliations:** Discovery and Translational Sciences Department, Faculty of Medicine and Health, Leeds Institute of Cardiovascular and Metabolic Medicine, University of Leeds, Leeds, UK; School of Biology, Faculty of Biological Sciences, University of Leeds, Leeds, UK; Discovery and Translational Sciences Department, Faculty of Medicine and Health, Leeds Institute of Cardiovascular and Metabolic Medicine, University of Leeds, Leeds, UK; Discovery and Translational Sciences Department, Faculty of Medicine and Health, Leeds Institute of Cardiovascular and Metabolic Medicine, University of Leeds, Leeds, UK; Discovery and Translational Sciences Department, Faculty of Medicine and Health, Leeds Institute of Cardiovascular and Metabolic Medicine, University of Leeds, Leeds, UK; Discovery and Translational Sciences Department, Faculty of Medicine and Health, Leeds Institute of Cardiovascular and Metabolic Medicine, University of Leeds, Leeds, UK; Discovery and Translational Sciences Department, Faculty of Medicine and Health, Leeds Institute of Cardiovascular and Metabolic Medicine, University of Leeds, Leeds, UK; School of Biology, Faculty of Biological Sciences, University of Leeds, Leeds, UK; Discovery and Translational Sciences Department, Faculty of Medicine and Health, Leeds Institute of Cardiovascular and Metabolic Medicine, University of Leeds, Leeds, UK

**Keywords:** developmental origins of health and disease, female reproductive tract, ovary, pregnancy, uterus

## Abstract

Environmental stressors to which a fetus is exposed affect a range of physiological functions in postnatal offspring. We aimed to determine the in utero effect of steroid hormones on the reproductive potential of female offspring using a porcine model. Reproductive tracts of pigs from female-biased (>65% female, n = 15), non-biased (45–54.9% female, n = 15), and male-biased litters (<35% females, n = 9) were collected at slaughter (95–115 kg). Ovaries and uterine horns were processed for H&E or immunohistochemistry. Variability of data within groups was analyzed with a Levene’s test, while data were analyzed using mixed linear models in R. In the ovarian reserve, there was a significant birth weight by sex ratio interaction (*P* = 0.015), with low birth weight pigs from male-biased litters having higher numbers of primordial follicles with opposite trends seen in pigs from female-biased litters. Sex bias held no effect on endometrial gland development. A lower birth weight decreased the proportion of glands found in the endometrium (*P* = 0.045) and was more variable in both male-biased and female-biased litters (*P* = 0.026). The variability of primordial follicles from male-biased litters was greater than non- and female-biased litters (*P* = 0.014). Similarly, endometrial stromal nuclei had a greater range in male- and female-biased litters than non-biased litters (*P* = 0.028). A crucial finding was the greater variability in primordial follicles in the ovaries from females derived from male-biased litters and stromal cell count in the endometrium of females from male- and female-biased litters. This could be inflating the variability of reproductive success seen in females from male-biased litters.

## Teaser

Females gestated in utero in extremes of sex ratios have altered ovarian reserve postnatally but not uterine capacity.

## Graphical Abstract

**Graphical Abstract:**
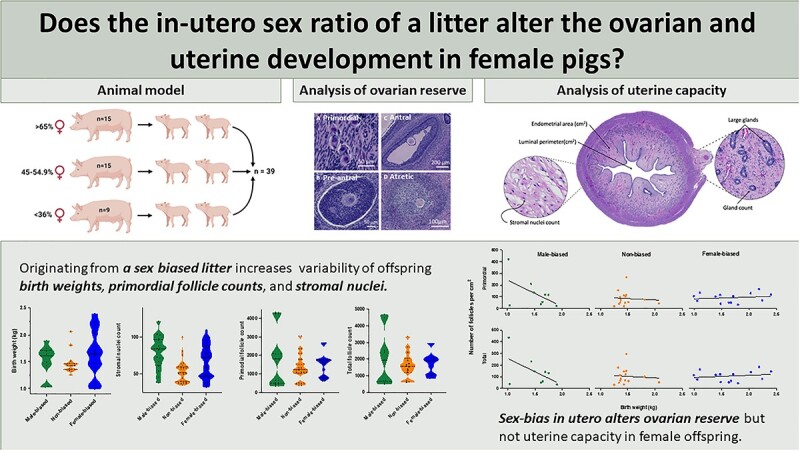

## Introduction

The maternal intrauterine hormonal environment has been shown to influence many aspects of offspring development, some of which are sexually dimorphic in nature [1]. Studies specifically investigating the influence of fetal hormones have indicated effects such as endocrine alterations [2], altered physiological development [3], and behavioral changes [4, 5]. These effects are observed in litter bearing species following an androgenization of the uterine environment, often caused by disproportionate numbers of males in utero. This may be a consequence of females positioned between males (which is proportionately more likely to happen in male-biased litters) or the overall proportion of males producing testosterone following sexual differentiation [6]. A biased litter, i.e., one that skews toward a predominantly androgenized or estrogenized environment (those consisting of >60% of one sex), occurs frequently both within commercial production systems and among wild pig populations, where proportions of 1.3:1 males to females per litter have been reported [7]. In the commercial pig, a sex biased in utero environment has been shown to affect several aspects of reproductive function in the offspring. Specifically, females that originate from male-biased litters have fewer teats [8], a lower conception rate at first mating [9], increased sensitivity to gonadotropins [10], and altered lutenizing hormone (LH) surge profiles [2], compared to offspring originating from female-biased litters. The cause of these changes in reproductive efficacy remains unclear.

Arguably, the most vital organizational event in female reproductive development during gestation is the development of primordial germ cells (PGCs). The PGCs are originally derived from the proximal epiblast cells of pre-gastrulating embryos [11], i.e., prior to generation of the three primary germ layers. In pigs, these have been identified in the dorsal mesentery at embryonic day (ED) 18–20 which then migrate to colonize, forming the genital ridge at ED23–24 [12]. It is established that the PGC’s undergo epigenetic reprogramming over a period of several weeks, beginning at ED12 [13]. In the pig, meiosis, initiation, and formation of primordial follicles begin at ED48 and continue until 25 days post parturition [14]. Differentiation of the Wolffian duct in the male begins at ED26, at which point secretion of testosterone begins [15]. Hence, laying down and formation of the primordial follicles in female offspring occurs once testosterone from male littermates is present in the uterine environment [16]. However, it is currently unclear whether an androgenized uterine environment affects PGC formation or follicular recruitment of the PGCs in the pig.

In addition to PGC establishment and specifically ovarian reserve establishment, an additional factor that contributes to reproductive success is uterine capacity [17]. Ovulation and conception rates in the breeding sow can be greater than 95% [18], maximizing litter sizes requires reduction in the loss during the pre- and peri-implantation period of pregnancy [17–19]. This coincides with conceptus elongation, synthesis, and release of estrogen for maternal pregnancy recognition, and trophectoderm differentiation, which is followed by fetal and epithelial attachment [20]. There is then a secondary wave of embryonic loss at ED 30–40 due to crowded in utero conditions [21]. It is therefore clear that litter sizes, and conceptus survival, are contingent on uterine capacity. This is determined by three main factors: uterine length, uterine blood flow, and uterine gland development [21]. It is vital that the uterine endometrium can recognize and respond to maternal and conceptus signals crucial for pregnancy [21–23] but the uterine capacity will define the environment in which fetal development occurs [22]. The heterogeneous nature of the endometrium, composed of several different cell types including secretory cells such as the luminal and glandular epithelium [19, 24], facilitates maternal and fetal interactions. Endometrial glands along with the luminal epithelium secrete uterine luminal fluid, a complex array of proteins and related substances [25]. The uterine luminal fluid is critical for endometrial function and conceptus survival as it contains enzymes, growth factors, cytokines, nutrients, transport proteins, and other regulatory molecules (reviewed by [26]). Maternal endometrial gland hyperplasia and hypertrophy is extensive during gestation [27, 28] with large amounts of granular, acid phosphate-positive material within the glands, indicative of a high level of secretory activity [23]. Sheep with blocked uterine horn (UH) gland development (UGKO—Sheep Uterine Gland Knock Out) indicate a failure of conceptus elongation at ED14 and are rendered infertile [29]. Evidencing the fact that uterine glands are critical mediators for the uterine ability to support a successful pregnancy. Therefore, formation of adequate endometrial tissue is critical for pregnancy success [23, 27]. Although the glands form neonatally, the histogenesis of the initial UH development, and luminal epithelium, takes place in utero [23, 30] making this process vulnerable to environmental effects to which the fetus is exposed, including excess androgens and or estrogens as is observed in cows [23, 31–33]. Furthermore, in pigs, neonatal progesterone treatment initially accelerated gland development, but reduced adult glandular development [34]. Impairment, as described above, has been indicative of a reduced fertile capacity [30].

This study aims to investigate how a sex-biased in utero environment may influence the development of both the follicular pool and endometrial glands, both critical components for successful pregnancy. To do this, we investigated how gestation in litters with different sex ratios altered (1) follicular patterns and (2) uterine morphology and endometrial gland proliferation in the female offspring.

## Material and methods

After consultation with the University of Leeds Animal Welfare and Ethical Review Committee, no ethical approval was sought. It was deemed that due to no interventions taking place, and the pigs remaining fully under the commercial management, ethics was not necessary. This complies with the three Rs (replacement, reduction, refinement) strategy of utilizing animals which are already part of a process.

### Animal model

Our model recruited individual female offspring at birth that were retrospectively assigned to one of three experimental groups: females from (1) female-biased in utero litter (>65% females), (2) non-biased (45.9–55% females), or (3) male-biased (<35% females) litters. Only litters with 10 or more live-born piglets were included in the study, and mummified piglets were not included as accurate identification of sex was unreliable.

All animals were reared in a closed indoor system at the National Pig Centre on partially slatted floor systems. The pigs used were either not part of any other trials or used as control pigs in other dietary studies. These female pigs (JSR Large White x Landrace females JT dam-line x JSR Pietrain-based Geneconverter 900 sire-line) were tracked through the production system using RFID (Radio Frequency Identification) ear tags. The pigs were weighed weekly in the month leading up to slaughter in order to optimize an accurate estimate of slaughter weight. At commercial slaughter weight (95–115 kg), pigs were delivered to the abattoir weekly over three collection time points per batch, a total of 10 time points.

### Tissue collection and dissection

**Figure 1:**
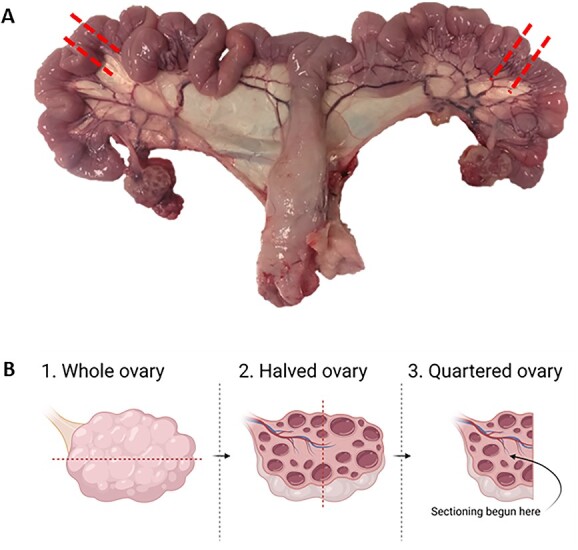
Schematic of how the reproductive tracts were dissected. (A) Uterine horn sections (1 cm) were taken at the marked points, and (B) ovaries were dissected, resulting in the final ovary sections. The place at which the first section was taken is marked out under 3. Quartered ovary. Created with Biorender.com.

To control for within-litter variability, two females were selected at random (using randomizer.org) for reproductive tract collection at slaughter from each litter. Reproductive tract collections occurred over three periods in November 2018, June–August 2019, and July–August 2020. The tracts were collected on the abattoir line and transported to the laboratory on ice within 1.5 h for tissue processing. The ovaries were dissected from the reproductive tracts and individually weighed. The right ovary was divided in half and placed in 10% neutral buffered formalin. The UHs were dissected away from the connective tissue and whole cross sections from one-third of the way down from the tip of the UH (Figure 1A) were also fixed in formalin for 48 h. Ovaries and UHs were removed from formalin and ovaries further divided in half lengthways through the cortex, resulting in a quartered ovary (ovary; Figure 1B). UH and ovary were subsequently dehydrated through a series of ethanol washes and imbedded in paraffin for histological examination. In total, the reproductive tracts from 39 piglets were assessed: male-biased = 9, non-biased = 15, and female-biased = 15. Unless otherwise stated all chemicals were obtained from Thermo Fisher Scientific (Waltham, MA, USA).

### Histomorphometry

#### Hematoxylin and eosin stain

For histomorphometry of the ovary, 8 μm serial sections of the embedded ovary from an individual were mounted on polylysine-coated Microslides (VWR International, Radnor, USA). For UH two individual slides containing whole cross sections of UH per animal were assessed. Slides were de-paraffinized three times in Histo-clear for 10 min. Slides were then hydrated in decreasing concentrations of ethanol (3 x 100% EtOH, 2 x 95% EtOH, and once in 70% EtOH). All slides were finally rinsed three times in tap water. Slides were incubated in hematoxylin (Sigma Aldrich, St. Louis, USA—7 g/L) for 5 min, slides rinsed in three times in tap water, dipped in 0.25% acid alcohol for 5 s, and immersed in cold water. At this stage, stain intensity was checked under a microscope before proceeding. Slides were washed in hot tap water for 1 min and then rinsed three times in cold tap water. Slides were then placed in a mordant of 95% EtOH. Slides were then stained in eosin for 10 min, and dehydrated through two changes of 95% EtOH, and three changes of 100% EtOH. Finally, slides were places in Histo-clear for 10 min three times. Ten serial sections per ovary per animal (each 160 μm apart) were selected with the initial section being closest to the ovarian cortex (Figure 1B). Serial sections for analysis were selected to be 160 μm apart to ensure an oocyte/follicle was not counted twice (as oocyte diameter is <110 μm [35]). For each individual one section per UH was also stained.

#### Immunohistochemistry—proliferating cell nuclear antigen

For investigating uterine gland proliferation, one section per UH were stained using an immunohistochemistry (IHC) technique using the Vector Lab VectaStain Elite ABC-HRP kit. Overnight incubations (4°C) of the sections were carried out with mouse monoclonal anti-proliferating cell nuclear antigen (PCNA) (1:200, Catalog number: MA511358; Thermo Fisher). Control sections were incubated with a mouse IgG Isotype Control (1:200, Catalog number: 31903; Thermo Fisher). The sections were then incubated with the secondary antibody and ABC reagent previously described for 60 min in a humidity chamber. Development of sections was carried out using diaminobenzidine substrate (Thermo Fisher).

### Image analyses

#### Ovarian reserve

**Figure 2:**
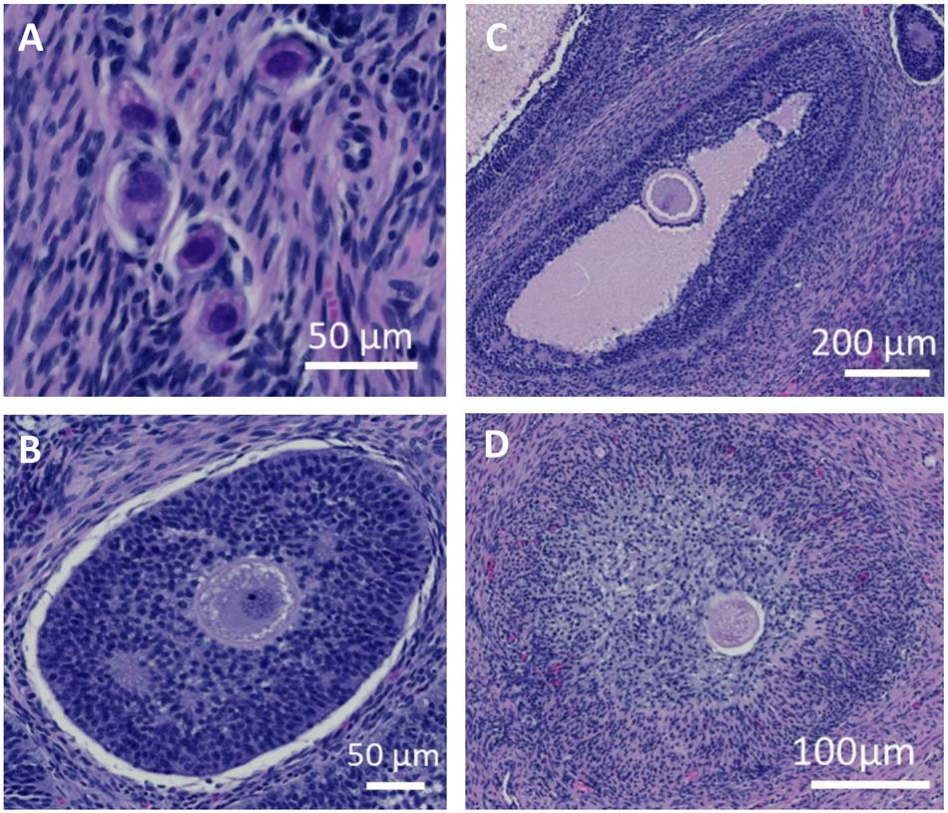
Images taken of follicles representing the different stages of development. (A) A cluster of primordial follicles. (B) Pre-antral follicle. (C) Antral follicle. (D) Atretic follicle.

To assess the ovarian reserve, the number of primordial, pre-antral, antral, and atretic follicles was counted and classified according to Almeida and colleagues (Figure 2) [36]. Primordial follicles (Figure 2A) were identified as an intact oocyte surrounded by a single layer of squamous (pre) granulosa cells. An enlarged oocyte that is surrounded by a single, or multiple, layers of cuboidal granulosa cells was classified as pre-antral (Figure 2B). Once the follicle had developed a clear antral cavity that was the same size as, or larger than, the oocyte, it was classified as an antral follicle (Figure 2C). The antral follicles have several layers of granulosa cells and have a well-developed thecal layer. All the above follicle types had intact oocytes with no signs of apoptosis or degradation as described by Almeida and colleagues [36]. If degenerative changes had occurred, including reduction of the oocyte or condensation of the nuclear chromatin, or changes to the antral cavity such as scattered granulosa cells, the follicle was identified as atretic (Figure 2D).

#### Gross uterine morphology and cell proliferation

**Figure 3:**
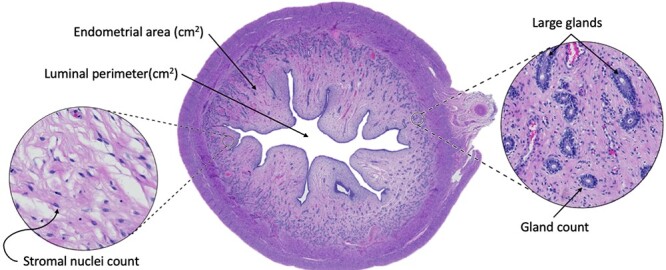
Images taken of uterine horn cross-sections. Example of uterine cross section with an H&E stain and indicators of the morphological structures investigated.

**Figure 4:**
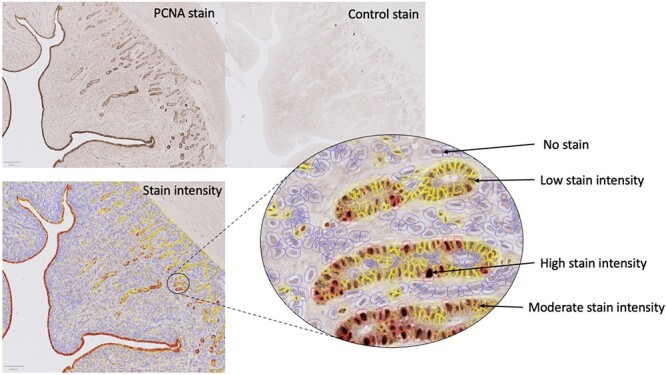
Images of IHC stained sections (8 μm) using a proliferating cell nuclear antigen antibody, including the identified stain intensity and the control section. The automated stain detection output can be seen including visual representations of cells considered to have no stain, low stain, moderate stain, and high stain intensities.

To assess endometrial capacity, we measured gross morphological structures. As a proxy measure of uterine support, we analyzed the ratio of secretory cells (luminal and glandular epithelia) to stromal cell area. The surface area of the endometrium per section was measured (cm^2^) along with the perimeter of the luminal gap (cm^2^). Manual counts were made of the glands in the endometrium of total sections, with the number of larger glands also counted. An automated cell nuclei detection within QuPath was used to count the stromal cells within an area of 20 000 μm^2^. These structures are visualized in Figure 3. Proliferative capacity of the endometrium was measured using the PCNA IHC stain. An automated DAB stain analysis was used for the entire sections of the UHs to measure stain intensity. This allowed for identification of cells with a negative or positive stain, along with division of positively stained cells into a low, moderate, or high stain intensity (Figure 4). Parameters used for the detection were refined using three sections previously stained in the stain optimization process. The parameters used for stain detection were as follows: pixel size, 0.5 μm with thresholds being low; 0.1–0.3, moderate; 0.3–0.5, high: 0.8–1.

### Data processing and analysis

All statistical analyses were performed in RStudio [37] using *lme4* [38], and data were plotted using PRISM (GraphPad Prism version 9.0.0 for MacOS, GraphPad Software, San Diego, CA, USA, www.graphpad.com). Normality was assessed using appropriate tests as detailed below, alongside histograms, and QQ-plots. Data considered to fit a Gamma distribution were tested using “gamma_test” in package “goft.” Collinearity between predictor values was checked using the “vif” function in R package “car.” Ovary weight (g) and slaughter age (days) was removed due to collinearity (>3). Critical alpha level was applied as *P* = 0.05. Akaike Information Criterion (AIC) model selection was used to distinguish between a set of possible models, each describing the relationship between the predictor variables. The AIC of the final model used for each analysis is detailed in Tables 1 and 2. Transformations were required for certain variables to fit their distributions. This was with either a square root transformation, function “sqrt,” or a log transformation, function “log” and is detailed in Tables 1 and 2.

**Table 1:** The response variables analyzed for the ovary and the specific model, model type, distribution of the variable, and AIC value of the most fitting model. All analyses were performed in RStudio and were carried out using *lme4*. Follicle numbers manually counted on H&E stained histological sections and reproductive tracts were collected from pigs originating from either female-biased (n = 15), non-biased (n = 15), or male-biased groups (n = 9)

Response variable	Model	Model type	Distribution	AIC value
Primordial follicles	1	GLMM	Gamma	369.0617
Recruited follicles	1	GLMM	Gamma	186.1362
Atretic follicles	2	GLMM	Gamma	190.557
Total follicles	1	GLMM	Gamma	371.1647

**Table 2:** The response variables analyzed for the uterine horn and the specific model, model type, distribution of the variable, and AIC value of the most fitting model. All analyses were performed in RStudio and were carried out using *lme4*. Reproductive tracts were collected from pigs originating from either female-biased (n = 15), non-biased (n = 15), or male-biased groups (n = 9). All observations were made from histological sections using either H&E stains or an IHC stain for proliferating cell nuclear antigen (PCNA)

Response variable	Model	Model type	Distribution	Transformation	AIC value
Rudimentary analysis					
Endometrial area (EA)	1	GLMM	Gamma		−127.258
Luminal perimeter (LP)	1	GLMM	Gamma	Log	−12.6513
Stromal nuclei (SN)	2	GLMM	Poisson		575.5295
Gland count (GC)	2	GLMM	Poisson		1398.412
Large gland count (LGC)	2	GLMM	Poisson		789.2035
IHC analysis					
Positively stained cells	1	GLMM	Gamma		−70.7763
Low stain intensity	1	GLMM	Gaussian		−121.477
Moderate stain intensity	1	GLMM	Gamma		−218.274
High stain intensity	1	GLMM	Gamma	Square root	−149.745
Proportional analysis					
LP: EA	2	GLMM	Gamma		1476.293
SN: EA	2	GLMM	Gamma		531.1063
GC: EA	2	GLMM	Gamma		1013.363
SN: LP	2	GLMM	Gamma	Square root	117.1338
GC: LP	2	GLMM	Gamma		749.575
GC:SN	2	GLMM	Gamma		749.575

The models used were one of the following (specified per response variable in Tables 1 and 2);

Models 1- *< Response variable >* per cm^2^ with predictor variables being *litter sex ratio* and *birth weight* as multiplicative, *slaughter weight* as additive, and *litter (ovary) or animal ID* nested within *litter (UH)* as random effect.

Models 2 - *< Response variable >* per cm^2^ with predictor variables *litter* as random effect, *litter sex ratio, slaughter weight* as additive, and *litter (ovary) or animal ID* nested within *litter (UH)* as random effect.

To investigate whether there was more variability within our investigated response variables between pigs of different in utero sex ratios, variance of data points was measured using “leveneTest” package.

#### Ovarian reserve

Data were analyzed in two ways. Firstly, looking at follicle numbers in the ovary as a whole, and secondly controlling for variations in the manual dissection of the ovary by investigating the number of follicles per cm^2^ of observed tissue. As results were reflective of each other, we have only reported the results per cm^2^ for reader ease.

Gamma regression models were used to test the effect of predictor values on the following response variables: (1) primordial follicle count, (2) recruited follicle count, (3) atretic follicle count, and (4) total follicular count. All predictor and response variables are described in Table 1.

#### Gross uterine morphology and proliferation

Analyses of data were performed to investigate difference of rudimentary morphology. Proportional analyses were conducted to investigate whether there was a difference in the proportion of secretory structures between bias litters. The following comparisons were made: luminal perimeter, stromal cells, and endometrial glands in relation to endometrial area. It was then important to compare whether the proportion of these secretory cells differed in proportion to each other between individuals from different in utero sex ratios. These proportional comparisons are all detailed in Table 2. The IHC stained cells were analyzed in the same manner as for the morphological analyses. However, low stain intensity was found to hold a normal distribution and was analyzed using model 2, and high stain intensity required a square root transformation. This is detailed in Table 2.

## Results

In total, 39 pigs were used for analysis, 15 females from female-biased litters, 15 from non-biased litters, and 9 from male-biased litters. Pigs were excluded from the trial if they failed to reach commercial slaughter, one pig was excluded once reproductive tracts were collected due to an active infection of the tract. The mean birth weight and slaughter weight (± SD) of the pigs were: (1) female-biased—1.65 kg (± 0.446) and 107 (± 8.140) kg, (2) non-biased—1.47 (± 0.207) and 110 (± 5.275) kg, and (3) male-biased—1.51 (± 0.303) and 113 (± 5.915) kg. No significant difference was observed. The ovarian tissue analyzed was on average 16.6 (± 3.197) cm^2^ in female-biased pigs, 17.8 (± 4.386) cm^2^ in non-biased pigs, and 15.0 (± 3.198) cm^2^ in male-biased pigs.

### Variability of reproductive parameters in individuals

**Figure 5:**
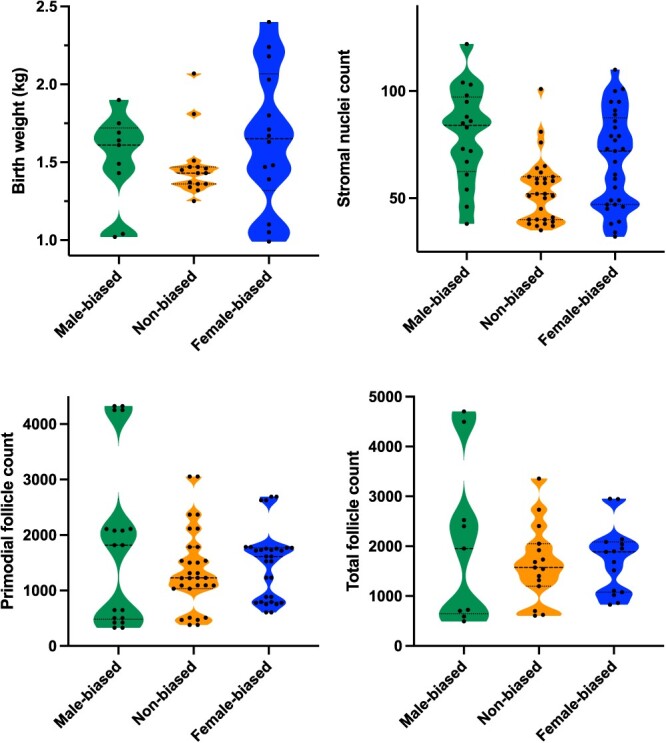
Weight and reproductive parameters from females gestated in different in utero environments. Variability of data represented by a violin plot of the birth weight (kg), stromal nuclei count, primordial follicles, and total follicles between male-biased (n = 9), non-biased (n = 15), and female-biased (n = 15) groups. Data were analyzed using a Levene’s test. Birth weights of piglets were measured on their first day postparturition after their first suckling event. Stromal nuclei count, primordial follicle counts, and total follicle counts were all analyzed on H&E stained sections. Manual counts were made for follicles, and an automate nuclei count used in QuPath 0.2.0.

The variability of data points between the sex ratio groups was analyzed as visualized in Figure 5. This revealed a larger range of birth weights (F-value = 4.073, df = 35, *P* = 0.026) from females originating in female- and male-biased litters compared to non-biased litters. There was a significantly larger variance of both primordial (F-value = 4.801, df = 36, *P* = 0.014) and total (F-value = 5.381, df = 36, *P* = 0.009) follicle numbers in females from male-biased compared to those that originated from non- or female-biased litters. An increased variability in the number of stromal cells in endometria recovered from females from sex-biased litters compared to non-biased litters was also observed (F-value = 3.8134, df = 71, *P* = 0.027). The variability did not differ between litter sex bias in endometrial area (F-value = 0.2472, df = 71, *P* = 0.7817), luminal perimeter (F-value = 2.3527, df = 71, *P* = 0.1025), nor total gland (F-value = 2.4973, df = 71, *P* = 0.08951) or large endometrial gland counts (F-value = 2.0419, df = 71, *P* = 0.1373).

### Interaction of birth weight and slaughter weight

The following results from the mixed models are summarized in Table 3.

**Table 3:** Mixed model outputs for each response variable. Outputs from the models run for each response variable including the number of animals in the analyses, specific test statistic, and *P*-value. Signif. Codes: 0 “^*^^*^^*^”; 0.001 “^*^^*^”; 0.01 “^*^”; 0.05 “.”

Response variable	No of animals/df	Interaction		Litter sex bias		Birth weight		Slaughter weight	
		Test statistic	*P*-value	Test statistic	*P*-value	Test statistic	*P*-value	Test statistic	*P*-value
Primordial follicles	34	2.637	0.008^*^	NA	NA	NA	NA	0.541	0.589
Recruited follicles	34	0.789	0.436	0.442	0.679	−0.669	0.509	0.995	0.330
Atretic follicles	34	NA	NA	0.817	0.414	−1.775	0.076	1.468	0.142
Total follicles	34	−2.754	0.006^*^	NA	NA	NA	NA	−2.754	0.611
Endometrial area (EA)	64	−1.416	0.157	0.022	0.825	−1.376	0.169	0.787	0.431
Luminal perimeter (LP)	64	−1.595	0.1107	1.096	0.2731	−1.915	0.055	−0.151	0.880
Stromal nuclei (SN)	64	NA	NA	−0.287	0.774	−0.916	0.359	1.237	0.216
Gland count (GC)	64	NA	NA	0.117	0.907	0.058	0.954	0.54	0.957
Large gland count (LGC)	64	NA	NA	−1.033	0302	0.432	0.666	−0.908	0.364
LP:EA	64	NA	NA	0.874	0.382	−0.142	0.887	−1.397	0.162
SN:EA	64	NA	NA	0.355	0.723	0.941	0.347	−1.315	0.188
GC:EA	64	NA	NA	−0.423	0.673	2.001	0.045.	−1.427	0.153
SN:LP	64	NA	NA	−0.416	0.667	1.919	0.055	−0.912	0.362
GC:LP	64	NA	NA	−1.315	0.188	1.709	0.087	0.045	0.964
GC:SN	64	NA	NA	−0.234	0.815	−0.634	0.526	0.767	0.443
Positively stained cells	63	−1.266	0.206	0.010	0.992	0.902	0.367	−0.965	0.335
Low stain intensity	63	0.713	0.482	0.225	0.800	−0.496	0.624	0.020	0.984
Moderate stain intensity	63	−0.853	0.394	−0.497	0.619	1.056	0.291	−0.629	0.529
High stain intensity	63	−1.533	0.125	0.204	0.839	0.737	0.461	−0.453	0.651

#### Follicular counts

**Figure 6:**
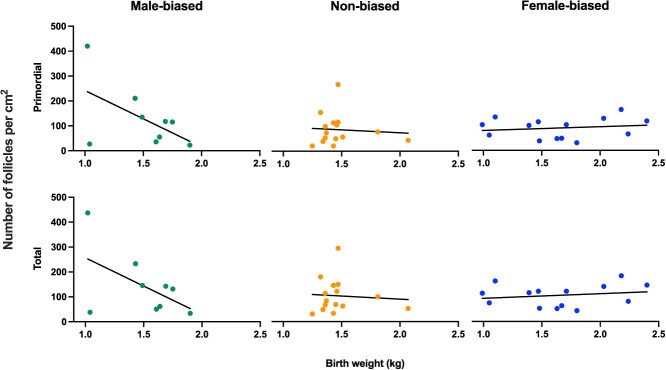
Birth weight of piglets for both primordial and total follicle numbers per cm^2^. Scatter plots hold fitted regression lines, individual points have been grouped according to the bias of the litter as either female-biased (>65% females), non-biased (45–54.9% females), and male-biased (<35% females), n = 34. Pigs from male-biased litters held a higher follicle count (both primordial and total) when they held a lower birth weight.

For an individual there was no significant interaction with in utero sex ratio of that piglet’s litter and their birth weight for the number of recruited follicles (GLMM; t-value = 0.789, n = 34, *P* = 0.436). There was a significant interaction in primordial (gamma GLMM; t-value = −2.637, n = 34, *P* = 0.008) and total (gamma GLMM; t-value = −2.754, n = 34, *P* = 0.006) follicle numbers per cm^2^ and sex ratio in utero. As seen in Figure 6, increasing birth weight was negatively correlated with primordial and total follicle numbers in females from male-biased litters. In contrast, female-biased and non-biased litters showed no difference in follicle numbers between different weights.

#### Uterine morphology and proliferating cells

There was no interaction observed between the sex ratio and birth weight for endometrial area (gamma GLMM; z-value = −1.530, n64, *P* = 0.126), nor luminal perimeter (gamma GLMM; z value = −1.595, n = 64, *P* = 0.1107). No significant interaction was found between birth weight nor sex ratio for the proportion of positively PCNA-stained nuclei (gamma GLMM; z-value = −1.266, n = 63, *P* = 0.206), nuclei with low (GLMM; z-value = 0.713, n = 63, *P* = 0.482), moderate (gamma GLMM; z-value = −0.819, n = 63, *P* = 0.413), or high nuclei stain intensity (gamma GLMM; z-value = −1.435, n = 63, *P* = 0.151).

### Non-interactive effects of sex ratio, birth weight, and slaughter weight

#### Follicular counts

No significant difference in numbers of recruited (GLMM; t-value = 0.442, n = 34, *P* = 0.679), nor atretic (gamma GLMM; t-value = 0.817, n = 34, *P* = 0.414) follicles were observed between sex ratios. Similarly, both recruited (GLMM; t-value = 669, n = 34, *P* = 0.509) and atretic (gamma GLMM; t-value = 1.775, n = 34, *P* = 0.0759) follicle numbers were not affected by the birth weight of an individual. The slaughter weight had no effect on any aspect of follicle count measured (primordial—gamma GLMM; t-value = 860, n = 34, *P* = 0.589; recruited—GLMM; t-value = 0.995, n = 34, *P* = 0.330; atretic - gamma GLMM; t-value = 1.468, n = 34, *P* = 0.142);, nor total—gamma GLMM; t-value = 2.754, n = 34, *P* = 0.611).

#### Uterine morphology

The sex ratio of the litter from which a pig originated, birth weight, or slaughter weight did not significantly affect the total cross section of endometrial area (gamma GLMM, n = 64; z-value = 0.140, *P* = 0.889; z-value = 1.289, *P* = 0.197; and z-value = 0.716, *P* = 0.474, respectively), luminal perimeter (gamma GLMM, n = 64; z-value = 1.096, *P* = 0.273; z-value = 1.915, *P* = 0.055; z-value = −0.151, *P* = 0.880, respectively), nor number of stromal cells as measured by nuclear staining (Poisson GLMM, n = 64: z-value = 0.346, *P* = 0.729; z-value = 0.058, *P* = 0.458; z-value = −0.559, *P* = 0.576, respectively).

The total number of endometrial glands (Poisson GLMM, n = 64; z-value = 0.117, *P* = 0.907; z-value = 0.058, *P* = 0.954; z-value = 0.054, *P* = 0.957, respectively) and larger glands alone (Poisson GLMM, n = 64; z-value = −1.033, *P* = 0.302; z-value = 0.432, *P* = 0.666; z-value = −0.908, *P* = 0.364, respectively) were not significantly different between individuals of different in-utero sex ratios, birth weights, or slaughter weights.

#### Proportional analyses

**Figure 7:**
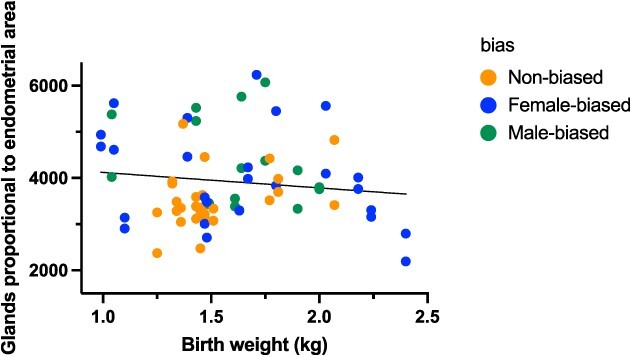
The ratio of glands to endometrial area in female pigs in relation to their birth weight (kg). Ratio given for those individuals gestated in either female-biased (>65% females), non-biased (45–54.9% females), and male-biased (<35% females), n = 34 in total.

To investigate whether the structures differed between sex ratio litter individuals in regard to the size of their reproductive tracts, the proportion of secretory structures relative to endometrial area were analyzed. No effects were seen for the ratio of luminal perimeter (cm^2^) nor stromal cell number nuclei to endometrial area for females from different sex ratio litters (gamma GLMM, n = 64; z-value = 0.843, *P* = 0.399; z-value = 0.371, *P* = 0.710, respectively), birth weight (gamma GLMM, n = 64; z-value = −0.099, *P* = 0.921; z-value = 0.836, *P* = 0.403, respectively), nor slaughter weight (gamma GLMM, n = 64; z-value = −1.387, *P* = 0.166; z-value = −1.004, *P* = 0.315, respectively). However, although the ratio of the total number of glands in the endometrial area for females from different sex ratios (gamma GLMM; z-value = −0.420, n = 64, *P* = 0.675) and slaughter weights (gamma GLMM; z-value = −1.422, n = 64, *P* = 0.155) were not significantly different, there was a significant difference in effect of the birth weight (gamma GLMM; z-value = 2.005, n = 64, *P* = 0.045) (Figure 7).

The analyses investigating the ratio of different secretory tissues against each other within the UH demonstrated no significant differences in ratios between stromal nuclei and luminal perimeter (gamma GLMM, n = 64; sex ratio—z-value = −0.294, *P* = 0.769; birth weight—z-value = 1.674, *P* = 0.094; slaughter weight—z-value = −0.552, *P* = 0.581), gland counts and luminal perimeter (gamma GLMM, n = 64; sex ratio—z-value = −1.315, *P* = 0.189; birth weight—z-value = 1.709, *P* = 0.087; slaughter weight—z-value = 0.045, *P* = 0.964), nor gland counts and stromal nuclei (gamma GLMM, n = 64; sex ratio—z-value = −0.434, *P* = 0.664; birth weight—z-value = −0.265, *P* = 0.791; slaughter weight—z-value = 0.314, *P* = 0.754).

#### Proliferating cells

The sex ratio of the litter from which a pig originated, birth weight, or slaughter weight did not show a significant effect on the proportion of PCNA-positive stained cells (gamma GLMM, n = 63; z-value = 0.015, *P* = 0.988; z-value = 0.992, *P* = 0.357; z-value = 0.934, *P* = 0.350; respectively), nuclei with a low stain intensity (GLMM, n = 63; z-value = 0.255, *P* = 0.800; z-value = −0.469, *P* = 0.624; z-value = 0.020, *P* = 0.984; respectively), moderate stain intensity (gamma GLMM, n = 63; z-value = −0.518, *P* = 0.604; z-value = 1.065, *P* = 0.287; z-value = −0.565, *P* = 0.572; respectively), or high stain intensity (gamma GLMM, n = 63; z-value = 0.023, *P* = 0.982; z-value = 1.043, *P* = 0.297; z-value = −0.162, *P* = 0.871; respectively).

## Discussion

The aim of this study was to investigate how gestation of individuals in a sex-biased in utero environment altered development and reproductive potential of the female offspring. We hypothesized that gestation of a female in a predominantly male or female in utero environment would alter primordial follicle pool and the development of the uterine capacity.

Irrespective of whether the follicle count was assessed on a per ovary or tissue size basis, the primordial follicle pool and total follicle count in the ovary were significantly more variable when females originated from a male-biased in utero environment. We also found that the birth weight affected the numbers of follicles present in an individual ovary in a sex-biased manner, i.e., different in those from a male-biased versus a female-biased litter. Previous research in litter bearing species including mice and pigs suggests an important role for androgens on follicle and corpus lutea (CL) development. In gilts this is illustrated by increased ovulation rates following dihydrotestosterone treatment [39], and CL dysfunction that is marked by decreased progesterone production when flutamide was used to block androgenic actions [40]. Overall, this study found no effects of sex ratio on the non-ovulatory recruitment, nor atresia of follicles, suggesting that an androgenized uterine environment does not interfere with non-cyclic folliculogenesis, nor the breakdown of follicles in the pre-pubertal commercial pig. Androgens in sheep were found to increase follicular recruitment when offspring were in a hyperandrogenized maternal circulation, resulting in early cessation of cyclicity [41]. However, it is important to note that sheep normally only bear one to three offspring per gestation and hence are not litter bearing. Therefore, they are less likely to be exposed to a biased uterine environment than in the pig. This may contribute to the species-specificity observed on androgenization. Additionally, the differences in functionality of different placenta types may well contribute to different results between species.

Despite no effects seen in the number of recruited follicles, females from male-biased litters had a higher total and primordial follicle count per cm^2^ when the individual pig held a low birth weight. However, females with a higher birth weight were found to have a higher number of primordial and total follicles if from female-biased litters. This suggests that the effect of an androgenized environment and an estrogenized uterine environment seems to have opposite influences on the development of the primordial follicle pool. This is further affected by the birth weight of the particular individual. An androgenized uterine environment may increase primordial germ cell proliferation, resulting in a larger total ovarian reserve in accordance with Seyfang and colleagues [42] who found that androgenized female pigs were more likely to ovulate and had higher CL counts when from a male-biased compared to female-biased litters, when treated with gonadotrophins at 18 weeks of age. Research in non-litter bearing species, who will hold different timings of PGC establishment, conflicts with that in pigs; findings in sheep in which individuals from a hyperandrogenized uterine environment displayed lower total ovarian reserve than their control counterparts [41]. This may be due to the pre-mentioned species differences, or due to the study design (intramuscular testosterone propionate in pregnant ewes, rather than uterine hyperandrogenization). This was an unexpected finding, as piglets that are below 1.3 kg at birth have been found to exhibit less competent postnatal development and reduced survivability [43], suggesting a detrimental effect of low birth weight on offspring. Compensatory growth following low birth weights has been found to lead to delayed puberty onset in mice [44]. Low birth weight piglets have also been found to grow less than normal and high birth weight piglets throughout their life course [45] and do not display the “catch-up” that piglets that have been fed a restricted diet do [46].

Despite there being, to our knowledge, no previous research specifically investigating the effect of an in utero sex bias on the uterine morphology or efficiency, postnatal uterine gland development is influenced by lactocrine aspects [47] and estrogenic influences leading to reduced uterine responsiveness to embryotropic signals [30]. Contrary to our hypotheses we did not find any evidence suggesting that gestation of a female in a male-biased uterine environment is detrimental to uterine development in our study. We did not demonstrate any effect of litter sex ratio on any of the defined uterine measures, their ratios, or the cell proliferation. However, it was found that the birth weight of a female pig had a significant effect on the proportion of glands in the uterus, relative to the uterine size. In this instance, the higher the birth weight, the lower the proportion of glands. This may be due to a larger uterus rather than specifically lower developed uterine glands. What is known is that females from male litters reach puberty at an older age [48] and have lower LH surges in gilts [42]. However, gilts from male-biased litters were more likely to ovulate in a cycle and had higher numbers of CL than those from female-biased litters [42]. There was a clear negative effect of originating from a male-biased litter on the reproductive success of female offspring. What is clear is future studies will need to investigate specific components of embryo loss in females that are gestated in a predominantly male environment. We propose that female pigs should be selected at specific points of cyclicity to further investigate whether there are effects on Cl numbers and or size to understand if this effect occurs in reproducing gilts and sows.

The most important outcome that we found in this study is the increased variability in reproductive measures in females originating from male-biased litters. In mice, the strain has shown to account for major variation with regard to follicular profiles [49] and great variability in ovarian reserve of individuals through the neonatal period and puberty [50, 51]. The variation in follicle number is considerably smaller in both non- and female-biased litters. There may be an underlying cause of the increased variation of total ovarian reserve in females from male-biased litters that hold functional consequences not yet understood. What is also evident is that the number of stromal cell nuclei within the endometrium was significantly more variable in females that came from either extreme, male- and female-biased litters. This variability in females from male- and female-biased litters was also seen in the birth weight of pigs. This could be caused by variation in the neighbors in utero, i.e., variation because females were gestated between two females, two males, or one male and female. This could potentially impact on stromal-derived growth factors that enhance secretion from the luminal and glandular epithelium [52]. The stromal cells are also key in supporting the underlying implantation structures and hence [52], variability within their numbers could lead to variability in uterine implantation capabilities and pre- and peri-implantation survival of the embryo/conceptus. Variability is known to be a major issue for pig producers and is commonly found in many different aspects of production [53]. Our data suggest that a biased litter may be a contributor to the variability of reproductive output commonly reported in female pigs. Furthermore, such biased litters were found to lead to more variation in the birth weight of offspring. Selection for larger litter sizes over time has resulted in litters of higher numbers but with low and greatly variable birth weights [54]. Low birth weight piglets from large litters are often cross fostered or euthanized as they will not be able to compete with their larger siblings for teats and have poor pre-weaning survival rates [21].

Under the premise of this study, pigs were expected to, at slaughter, not yet have become cyclic. Some pigs may however have reached puberty earlier than the norm, skewing results. Therefore, this suggests that to truly understand the effects of a biased litter on the recruitment and atresia of follicles, future work should utilize naturally synchronized gilts that have been monitored for ovarian activity, and these current findings should be cautiously interpreted. There may be an increase in the depletion of ovarian reserve in older pigs, which would normally see a 70% decrease from E50 to 300 days after birth [55]. Finally, albeit currently a very novel avenue, there may be an effect of an androgenized uterine environment on the development of oogonial stem cells, if they functionally exist within the pig. Synchronization would allow for a detailed understanding of deviating recruitment patterns observed in previous studies, which this experimental design does not hold sensitivity to fully investigate. Further to this study, investigating the depletion of the ovarian reserve over time would help understand the long-term effects that a bias may hold on reproductive longevity.

In conclusion, females that originate from a predominantly male-biased litter, i.e., an androgenized uterine environment, and have low birth weight, display increased primordial ovarian reserve along with increased variability of primordial follicle numbers as compared to pigs that originate from non- and female-biased litters. Conversely, a higher birth weight resulted in a greater primordial ovarian reserve if the female pig originated from an estrogenized uterine environment. Pigs from either litter bias (male or female) were found to have a significantly higher variation in birth weight than if a pig originated from a non-biased litter. These data have implications for reproductive potential of females gestated in sex-biased in utero environments.

## Data Availability

All data are provided in the manuscript.
